# Academic–practice partnerships in public health workforce development in China: a comparative analysis of 12 purposively selected cases

**DOI:** 10.3389/fpubh.2026.1900270

**Published:** 2026-09-15

**Authors:** Wenting Li, Yimeng Ren, Zhuanzhuan Fan, Lin Zhu, Guoliang Ma

**Affiliations:** 1 Nanjing Center for Disease Control and Prevention, Nanjing, China; 2 Chinese Center for Health Education, Beijing, China

**Keywords:** academic-practice partnerships, governance resilience, institutional isomorphism, public health workforce development, resource dependence theory

## Abstract

**Background:**

Academic–practice partnerships (APPs) are increasingly used in public health workforce development, but evidence on their institutional drivers and varied development pathways in China remains limited. This study examines how institutional pressures, interorganizational resource relationships, and boundary-spanning practices are reflected in the development of APPs in China.

**Methods:**

An exploratory multiple-case comparative design was adopted. Twelve APP cases were purposively selected across Chinese regions with diverse resource endowments. Drawing on institutional isomorphism, Resource Dependence Theory (RDT), and boundary-spanning theory, framework analysis was used to compare policy documents, authorized internal institutional records, and publicly available materials across documentary sources.

**Results:**

Cross-case analysis identified four forms of integration and boundary-spanning: institutional arrangements, spatial or practice-setting integration, collaborative training mechanisms, and educational-to-professional development pathways. Dual-mentor arrangements were a recurring mechanism in the documentary evidence. Variation among cases was observed in resource strategies, driving forces, and stated training objectives, including technology integration versus contextual resource activation, administratively initiated versus demand-driven development, and research-oriented versus applied workforce preparation.

**Conclusion:**

APPs within China’s public health workforce development constitute dynamic institutional arrangements shaped by macro-level institutional pressures, meso-level resource interdependence and collaborative governance, and micro-level boundary-spanning practices. Sustaining these partnerships appears to require institutionalized governance structures, competency-oriented training systems, and incentives that move collaboration beyond formal alignment toward substantive integration.

## 1 Background

Since its establishment in the 1940s, public health and preventive medicine in China has historically faced a relatively disadvantaged position within the broader medical education system. According to P. G. Altbach’s center–periphery theory, the marginalization of a discipline may influence educational investment, professional development opportunities, and ultimately the capacity of the public health workforce to support health system development and governance (1, 2). Historically, public health education in China was strongly shaped by the Soviet model, which emphasized the training of highly specialized personnel for disease prevention and control. This model contributed to a persistent structural “clinical–preventive divide” in medical education (3), leading to long-standing professional identity challenges and retention difficulties among public health physicians (4, 5).

From a global perspective, the World Health Organization (WHO) has highlighted shortages and structural imbalances in the public health workforce (6) and called for reforms in education and workforce development (7). Academic–practice collaboration is one approach to linking education, research, and service. Academic Health Departments (AHDs), characterized by sustained collaboration between universities and public health agencies, provide an established example (8, 9). Following the COVID-19 pandemic, limitations of knowledge-oriented training have received renewed attention, alongside greater emphasis on competency-based and practice-oriented education (10–12).

Furthermore, public health workforce modernization is increasingly shaped by diverse and interdisciplinary challenges. Recent studies illustrate the expanding range of issues that public health professionals may encounter, including environmental exposures, climate-related systems risks, culturally grounded approaches to community well-being, and explainable artificial intelligence in health analysis (13–16). Although these studies do not evaluate APPs directly, they exemplify emerging domains that require environmental-health literacy, interdisciplinary systems thinking, community responsiveness, and responsible data use. Addressing these challenges requires training frameworks that integrate data analytics, digital literacy, and comprehensive community-health perspectives. Against this global backdrop, China has promoted reforms in medical education through the “New Medicine” initiative introduced in 2018 (17). This initiative calls for an integrated “bio-psycho-social-engineering-environmental” model (18) and stronger integration among medical education, scientific research, and healthcare practice (19–21). In response, diverse APP models have emerged across China as institutional approaches to public health workforce development.

Despite wider use of APPs, relevant international and Chinese literature can be organized into four overlapping areas: institutional governance, skills development, university–practice collaboration, and organizational mechanisms. Governance studies often emphasize macro-level policy and theoretical justification (22, 23), while skills research commonly examines specific pedagogical or competency-based approaches (24, 25). Many reports of university–practice collaboration are descriptive case accounts, which offer contextual detail but limited cross-regional comparability (26). Organizational adaptation to local resource conditions has also received less explicit theoretical attention. A comparative, multilevel analysis may therefore help connect policy context, organizational arrangements, and implementation mechanisms.

No single conceptual lens captures every level of APP implementation. This study therefore uses three complementary perspectives. Institutional isomorphism sensitizes the analysis to macro-level policy and regulatory pressures that may encourage similar organizational forms (27). Resource Dependence Theory (RDT) directs attention to meso-level resource interdependence and organizational strategy (28). Boundary-spanning theory focuses on micro-level roles and practices that may support knowledge exchange across organizational settings (29, 30). Together, these perspectives provide a multilevel framework for examining, rather than presuming, how policy pressures, resource relationships, and boundary-spanning practices are expressed across the cases.

This study makes two main contributions. First, it compares 12 purposively selected APP cases across heterogeneous resource settings to identify recurring and divergent organizational pathways. Second, it develops an integrated analytical framework linking macro-level institutional pressures, meso-level resource relationships and collaborative governance, and micro-level boundary-spanning practices. The framework is intended as a basis for analytical comparison and context-sensitive application beyond the selected Chinese cases.

## 2 Materials and methods

### 2.1 Case selection and purposive sampling strategy

Cases were selected through purposive maximum-variation sampling to support analytical generalization, consistent with Yin’s multiple-case study methodology (31). As shown in Figure 1, selection proceeded through four stages: identification of 45 collaborative programs, preliminary screening, detailed eligibility assessment, and purposive selection of 12 APP cases. The final cases were selected to maximize variation according to the following criteria:

(1) Regional and ecological variation: inclusion of highly developed coastal settings (e.g., Shanghai and Guangzhou) and inland western or northern settings with distinct public health contexts (e.g., Dali and Inner Mongolia).
(2) Institutional and operational integration: priority was given to programs with documented organizational structures or recurring operational mechanisms rather than memorandum-only collaboration. Partnerships restricted to isolated, disease-specific activities were not included in the detailed comparative sample.
(3) Data richness and accessibility: ensuring sufficient access to internal policies, training curricula, and multi-year evaluation reports.

**Figure 1:**
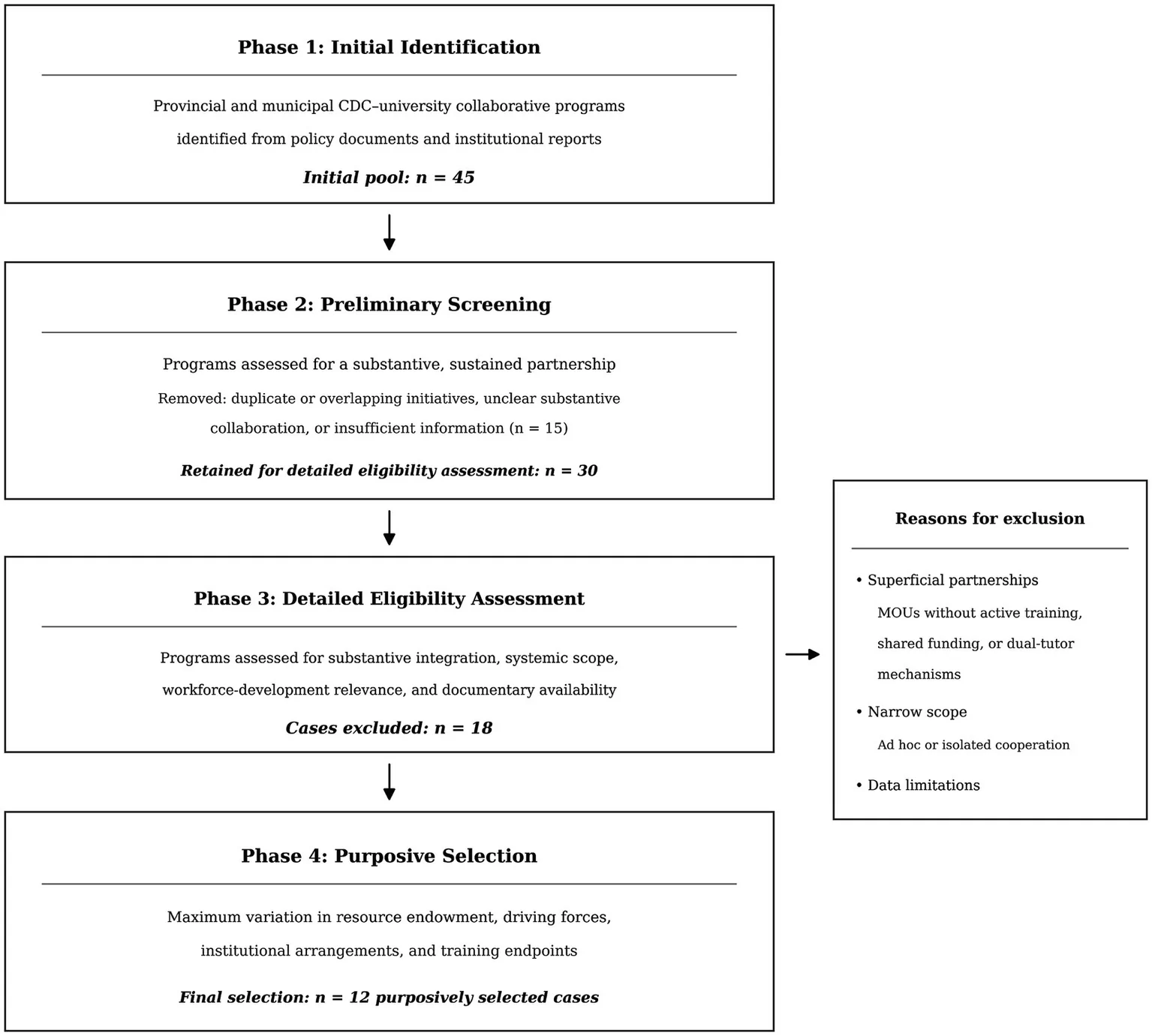
Flowchart of APP case selection. Of 45 CDC–university collaborative programs identified, 30 underwent detailed eligibility assessment and 18 were excluded because of superficial partnerships, narrow scope, or insufficient documentary evidence, yielding 12 purposively selected cases.

Based on these criteria, 12 APP cases were purposively selected across China (Table 1). The sample includes regional collaboration models in Shandong Province, Guangzhou, Tianjin, Inner Mongolia Autonomous Region, Chongqing, Shanghai, Hangzhou, and Dali in Yunnan Province, as well as institution-led practice networks involving Sichuan University, Capital Medical University, Nanjing Medical University, and Hebei Medical University. The selection intentionally emphasized established and information-rich partnerships to enable cross-case comparison and analytical interpretation across heterogeneous settings.

**Table 1:** Comparative synthesis of the 12 purposively selected Academic–Practice Partnership (APP) cases.

Case/region	Institutions involved	Type of partnership	Predominant resources	Collaboration mechanisms	Objectives pursued	Main results
1. Shandong	Shandong Provincial CDC and Shandong First Medical University	Entity co-construction	Government-coordinated university disciplines, provincial CDC platforms, and high-level biosafety laboratories	Co-construction of the college, reciprocal staff appointments, personnel integration, and dual-tutor system	Enhancing emergency response capacity and cultivating practice-oriented public health professionals	Established the “Four-Integration and Five-Innovation” model and a joint environmental medicine center; graduates rapidly become epidemic control backbones.
2. Guangzhou	Sun Yat-sen University and Guangzhou CDC	Platform integration	University academic resources, CDC real-world scenarios, and infectious disease big data	“Four-pillar” model (graduate school, experimental class, volunteer team, joint center), clinical-preventive-management rotational practice	Cultivating interdisciplinary professionals proficient in clinical medicine, public health, and management with decisive problem-solving capabilities	Innovated the “4 + N + Comprehensive Evaluation” system, jointly published high-impact SCI papers, and produced core policy guidance reports.
3. Tianjin	Tianjin CDC Authority and Tianjin Medical University	Strategic synergy driven by “Dual-High” construction	High-level public health school, regional CDC center advantages, and AI/supercomputing resources	Establishing a policy research center and feature laboratories, integrating residency training with MPH curricula	Cultivating high-end composite talents in “policy research + management practice” to translate academic outcomes into governance policies	Created “AI+” typical CDC application scenarios and led the formulation of multiple national standards and planning strategies.
4. Inner Mongolia	Inner Mongolia CDC & medical universities	Localized real-world teaching cooperation	Unique natural foci for endemic diseases (e.g., plague, brucellosis) in northern ecological barriers and biosafety labs	Deep pairing of teaching departments, joint research projects, and collaborative dual-tutor guidance	Cultivating applied talents familiar with ethnic region characteristics, equipped with field execution, data analysis, and laboratory testing skills	Built a plague control demonstration area and chronic disease big data platform; graduates quickly filled regional public health backbone vacancies.
5. Dali	Dali University & border prefecture/county CDCs in Yunnan Province	Regional collaborative alliance	Extensive borderlines and complex real-world scenarios for cross-border infectious disease control	Establishing the “National Border CDC Alliance” and employment communities with mutual expert appointments	Cultivating “border-serving, highly skilled, and retainable” applied public health practitioners	Established the China-ASEAN Public Health Technology Cooperation Sub-center, significantly enhancing international influence in cross-border public health.
6. Sichuan	West China School of Public Health (Sichuan University), local CDCs, and medical institutions	Industry-education integration	32 industry-education integration bases and real-world disaster rescue scenarios	“Supply–demand matching” project-driven mechanism, a pool of over 120 industry mentors, practical skills assessments in admissions, and disaster drills	Addressing the practice deficit and demand mismatch, and refining emergency response and cross-departmental coordination skills	Formed a “theory + skill + drill + practice” loop; demonstrated effectiveness in real-world field rescues such as severe mudslides.
7. Hangzhou	Hangzhou CDC and Zhejiang University (and other universities)	Teaching-oriented CDC model	Complex operational chains of a megacity and abundant real-world epidemiological investigation resources	Three-tier teaching management structure, modular rotational training, visual teaching materials, and bidirectional incentive evaluations	Deepening the “battle-teaching integration” to cultivate applied professionals with strong occupational competencies	Published the “Basic Practical Skills Operation Tutorial for Preventive Medicine” and achieved win-win school-local cooperation.
8. Beijing	Capital Medical University, affiliated clinical hospitals, and Beijing CDC	Tripartite joint education	Capital’s high-level clinical specialty resources and municipal/district CDC practice networks	“Basic-Clinical-CDC” three-stage linked training, parallel tracks for MPH and standardized residency training (four-certificate integration)	Cultivating high-end professionals with clinical-preventive integration, international vision, and major security safeguarding capabilities	Achieved a 100% pass rate in physician qualification exams, selected as a municipal industry-academia collaborative base, and developed national virtual simulation courses.
9. Nanjing	Nanjing Medical University and Nanjing CDC	Affiliated institute and training center co-construction	University academic platforms combined with municipal CDC operational bases	Co-constructing a public health research institute and integrated training center, adopting CBL/PBL teaching methods	Enhancing practical capabilities in emergency response, health monitoring, and big data analysis guided by competency	Built an integrated “teaching-learning-practice-management-evaluation” training system and a step-by-step cultivation program for young teachers.
10. Chongqing	Chongqing CDCs, clinical medical institutions, and Chongqing Medical University	Dual-track integration of standardized training and MPH	“1 + 5 + 6” standardized training resource network (core base, grade-A hospitals, and collaborative units)	Issued training management measures, fused training with core projects, and implemented a joint dual-tutor system	Breaking institutional entry barriers to cultivate high-quality talents who are “theory-savvy, practice-strong, and innovative”	Pioneered the “Excellent Public Health Physician Class,” issued pilot integration policies, and achieved a > 97% graduation pass rate in training.
11. Shanghai	Shanghai CDC and university/research institute think tanks	Project-driven science-education cultivation	Frontline pain points of CDC’s main responsibilities and top university academic think tanks	Implementation of the “Qinggeng Program,” one-on-one precise mentorship, full-process scientific research management, and ethical review	Overcoming the young talent deficit of “only working, not researching” and strengthening independent research capacity for applied translation	Solidified the “first mile” of talent cultivation, helping young CDC staff secure provincial and national research grants.
12. Hebei	Hebei Medical University and Hebei Provincial CDC	Continuous training and employment community	Provincial key laboratory clusters, large-scale public health training centers, and real-world public health projects	Cohesive undergraduate-master-doctoral training, “tutor + project” system, “early and frequent clinical exposure,” and specialized streaming in junior year	Aligning with big data governance needs, deepening strategic public health talent reserves, and enhancing career adaptability	Undertakes over 70% of provincial public health policy consulting, developed epidemic prediction models, and achieved high employment and satisfaction rates.

### 2.2 Data sources and triangulation across documentary sources

Information was compared across different categories of documentary sources to assess consistency between policy expectations, institutional arrangements, and reported training activities. Macro-level policy documents were used to identify institutional pressures; meso-level administrative records documented the formal establishment and governance of partnerships; and micro-level training and evaluation materials described reported implementation. Because all sources were documentary, this procedure was treated as source triangulation across policy, administrative, training, and evaluation materials. Documentary evidence was interpreted as evidence of documented implementation across the selected cases.

The document corpus comprised 112 documents produced between 2019 and 2025. On average, 8 to 12 documents were examined for each case, with national and provincial policy documents also informing more than one case where relevant. The corpus included publicly available sources and authorized internal institutional materials. Internal materials were used under institutional permission and are subject to confidentiality and third-party access restrictions. The materials were categorized into three source groups:

*Macro-level policy documents (21 items)*: Including national and provincial guidelines on public health reform and medical education (e.g., guidelines issued by the National Health Commission and local government implementation opinions).
*Meso-level institutional administrative records (42 items)*: Including strategic cooperation agreements, joint-establishment charters, and internal management protocols provided by participating CDCs and universities.
*Micro-level training and evaluation materials (49 items)*: Comprising training curricula, implementation details of the dual-tutor system, postgraduate practice assessment guidelines, and official program summaries.

### 2.3 Study design

This study adopts an exploratory multiple-case comparative design. According to Yin (31), case-study design is particularly suitable when research questions focus on “how” and “why,” and when the investigator cannot exert control over real-world events. Compared with single-case designs, multiple-case designs support analytic replication, enabling cross-case comparison to identify shared logics as well as divergent development pathways.

*Step 1: Data familiarization*. All original case materials and relevant policy documents from the 12 cases were reviewed in depth. Key information nodes were identified and extracted.

*Step 2: Development of the analytical framework*. Four *a priori* analytical dimensions were defined from the theoretical framework and research questions: institutional arrangements, spatial or practice-setting integration, collaborative training mechanisms, and educational-to-professional development pathways (32). These dimensions were translated into operational definitions, documentary indicators, and representative coding examples (Table 2).

**Table 2:** Operationalization of the four-dimensional analytical framework.

Dimension	Operational definition	Documentary indicators	Theoretical basis	Representative coding example
Institutional boundary-spanning	Formal integration of organizational authority, responsibilities, and governance across universities and public health institutions.	Joint institutes; signed co-construction agreements; reciprocal appointments; joint committees; shared management arrangements.	Institutional isomorphism; RDT	An institutional agreement documented reciprocal appointments of university and CDC personnel and formal joint governance.
Spatial boundary-spanning	Conversion of regional disease profiles, ecological conditions, and practice settings into shared training resources.	Field bases; endemic-disease sites; border-region rotations; regional CDC networks; access to operational facilities.	RDT; boundary spanning	Training materials described supervised field practice in regional disease-control settings and cross-institutional practice bases.
Mechanism boundary-spanning	Integration of academic instruction and frontline practice through joint supervision, curricula, assessment, and feedback.	Dual-tutor systems; joint curricula; field drills; CBME/EPA-aligned assessment; bidirectional mentor evaluation.	Boundary spanning; RDT	A training plan assigned complementary responsibilities to university and CDC mentors and linked assessment to field tasks.
Pathway boundary-spanning	Linkage of university education with professional entry, employment, and continuing workforce development.	MPH–residency integration; employment pathways; early-career mentoring; continuing education; longitudinal training sequences.	Institutional isomorphism; boundary spanning	Program records described an integrated sequence connecting degree education, practice training, credentialing, and employment development.

*Step 3: Indexing, coding, and analytical consistency*. Two researchers independently coded meaning units consisting of relevant sentences or paragraphs within the documentary corpus using the initial codebook. Coding discrepancies and proposed refinements were recorded in an analytic decision log and discussed during structured peer debriefing (33). Differences that could not be resolved by the two coders were reviewed by a third senior researcher. The codebook and case-by-dimension matrix were updated after consensus decisions. Consistent with the interpretive-consensus orientation of framework analysis, analytical rigor was supported through independent coding, documented decisions, third-researcher adjudication, source triangulation, and an auditable evidence matrix; a numerical agreement coefficient was not used as the primary consistency criterion.

*Step 4: Mapping and classification*. The coding results were organized through a two-stage mapping process (34). First, the four-dimensional framework was populated to construct a cross-case practice map. Second, a multidimensional matrix was used to compare heterogeneous development pathways. Table 2 shows how each analytical dimension was operationalized and linked to documentary indicators and anonymized coding examples.

*Step 5: Interpretation and synthesis*. The multilevel framework was used to interpret each case and compare patterns of similarity and variation across implementation pathways.

## 3 Results

### 3.1 Practice mapping and classification of Academic-Practice Partnership (APP) models

As shown in Figure 2, cross-case analysis of 12 purposively selected cases identified four boundary-spanning dimensions—institutional, spatial, mechanism-based, and pathway-based—through which APPs may develop from loosely structured collaboration toward substantive academic–practice integration. Cross-case synthesis further identified dual mentorship, competency-oriented training, and interdisciplinary platforms as recurring mechanisms.

**Figure 2:**
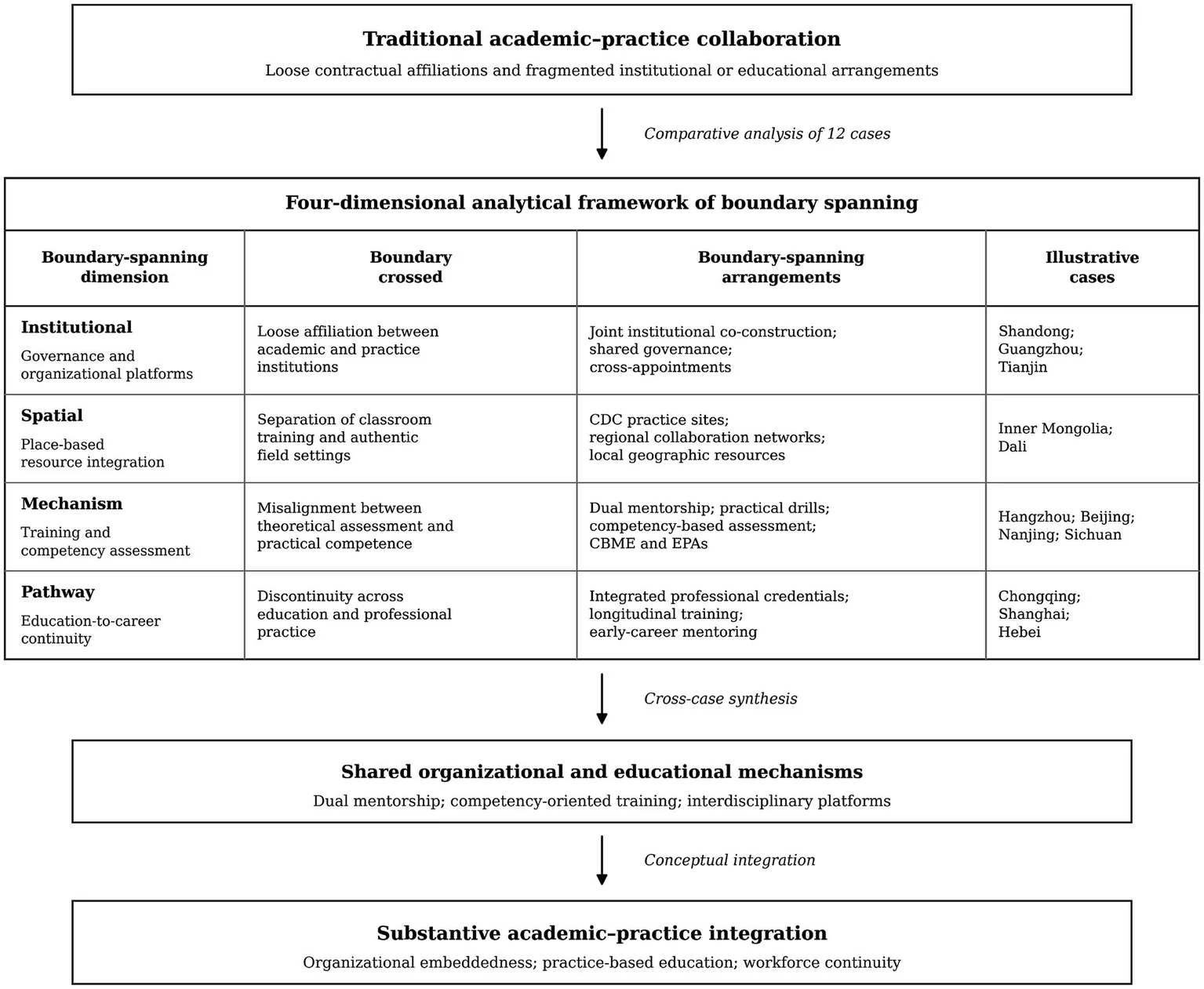
Four-dimensional analytical framework of boundary spanning in APPs. Comparative analysis of 12 cases identified institutional, spatial, mechanism-based, and pathway-based dimensions. Cross-case synthesis further identified dual mentorship, competency-oriented training, and interdisciplinary platforms as recurring mechanisms associated with substantive academic–practice integration.

#### 3.1.1 Institutional boundary-spanning and platform integration: from loose collaboration to entity-based co-construction

Driven by changing regional health-governance requirements and public health emergencies, loose contract-based collaboration may be insufficient to address fragmented governance challenges (35). The documents indicate that Shandong, Guangzhou, and Tianjin adopted more formalized, entity- or platform-based arrangements. Cross-case comparison nevertheless shows that their specific integration pathways varied according to local institutional priorities and available resources.

Shandong Province emphasized personnel and structural integration through top-down administrative coordination. Its formal co-construction agreement described a mechanism for connecting personnel, information, and resources through reciprocal professional appointments and cross-tenure leadership. The appointment of provincial CDC experts as university master’s supervisors illustrates formalized personnel integration and recurring placement of MPH students within CDC workflows.

In contrast, Guangzhou pursued programmatic and operational integration through a “Four-pillar” model comprising a Graduate School, a Medicine–Prevention–Management Experimental Class, a Volunteer Epidemiological Team, and a Joint Research Center. Program documents report the training of 122 MPH students across multiple cohorts and joint research outputs, including SCI-indexed papers with reported impact factors up to 32.1. These figures document program scale, sustained activity, and a pathway linking interdisciplinary training with joint research production.

Tianjin represents a model of strategic and technological integration associated with its “Dual-High” construction, combining a High-Level School of Public Health with a National Regional Public Health Center. The partnership established a Public Health Policy Research Center and used AI and supercomputing resources to support data analysis and policy simulation. Institutional records report the joint completion of more than 10 policy studies and participation in national AI application scenarios. These outputs illustrate a documented pathway from academic collaboration to policy-oriented work and technology-supported governance.

#### 3.1.2 Spatial boundary-spanning and resource integration: localized real-world teaching based on geographic advantage

In the western and border-region cases, geographic and epidemiological settings were incorporated into practice-based training (36). Inner Mongolia and Dali both used locally available settings and networks, but their documented strategies differed: the former emphasized ecological and laboratory resources, whereas the latter emphasized a cross-regional public health network. Geographic location was therefore treated as both a contextual constraint and a potential training resource.

The Inner Mongolia CDC drew on regional ecological and laboratory resources relevant to zoonotic diseases such as plague and brucellosis. By linking university teaching departments with CDC operational offices, the partnership used field settings and a biosafety level-3 laboratory as training resources. Program documents report more than 4,600 specialized instruments and the training of over 100 students. These indicators describe the scale of implementation and the integration of locally specific resources into training.

In contrast, Dali University, located in Yunnan Province, developed an external collaboration network serving communities across the province’s approximately 4,060-kilometer international border. Through the National Border CDC Alliance, the university collaborated with public health institutions across eight border prefectures and 31 county-level CDCs. Program records report 401 frontline experts serving as part-time instructors and more than 30 recent graduates entering border-area CDC positions. These figures illustrate the reach of the network, practitioner participation, and workforce placement in border-area public health institutions.

#### 3.1.3 Mechanism boundary-spanning and integration of medical education and practice: reconfiguring competency-based assessment

Connecting academic instruction with frontline practice requires explicit training and assessment mechanisms. The cases from Sichuan, Hangzhou, Beijing, and Nanjing documented different approaches, including field-based instruction, tripartite training, practical-skills assessment, and longitudinal evaluation. Together, these arrangements reflect competency-oriented approaches adapted to different training contexts.

At the operational level, Hangzhou CDC described a “teaching-oriented CDC” model that incorporated students into supervised epidemiological practice. A standardized visual manual and a bidirectional evaluation mechanism were used to connect routine workflows with instruction and to assess both students and mentors. These features show how the model connected routine epidemiological practice with structured teaching and reciprocal evaluation.

At the interdisciplinary level, Capital Medical University implemented a Basic–Clinical–CDC tripartite training model that linked MPH education with standardized residency training. Program records report a 100% pass rate in the national physician qualification examination among the relevant cohorts and designation as a municipal industry–academia collaborative base. These indicators describe strong cohort performance and institutional recognition within the documented program.

The Nanjing model uses a closed-loop competency assessment to evaluate multidimensional occupational capabilities across successive stages of training. Sichuan University likewise moved practical assessment earlier in the training pathway by including 10 core practical skills, such as personal protective equipment procedures and water sampling, in postgraduate entrance assessment and by involving students in documented disaster-response exercises. These arrangements illustrate mechanisms intended to narrow the gap between theoretical knowledge and practical execution.

#### 3.1.4 Pathway boundary-spanning and professional integration: life-course training for long-term workforce development

Pathway-based integration concerns how education is connected with professional entry and continuing development. The cases from Chongqing, Shanghai, and Hebei documented arrangements at different career stages, including credentialing, early-career mentorship, and longer-term training and employment pathways. Taken together, these cases illustrate institutional links spanning successive stages of professional development.

At the professional-entry stage, Chongqing linked standardized public health residency training with MPH education through a “1 + 5 + 6” network comprising one core CDC, five tertiary hospitals, and six collaborating units. Program records report a graduation-assessment pass rate above 97% across cohorts. This reported result is consistent with a structured pathway linking residency training and MPH education.

At the early-career stage, the Shanghai CDC established the Qinggeng Program, a project-based science–education initiative with one-to-one mentorship. Program records report 170 participants selected from 183 applicants across four cohorts; outputs from two cohorts included 88 papers and nine approved research grants. These results illustrate the research activity and mentoring outputs associated with the program.

For longer-term workforce development, Hebei Medical University documented an undergraduate–master’s–doctoral training and employment pathway. Practical teaching accounted for more than 35% of total academic hours, and specialized pathways began in the junior year. Institutional records report an average employment rate of 96.8%, a 91.3% field-match rate, and participation by collaborative teams in more than 70% of provincial public health policy consultations. These figures describe sustained outputs across education, employment, and policy participation.

### 3.2 Recurring elements across boundaries in collaborative education

Despite variation in resource endowments and implementation pathways, three recurring features were documented across several cases: formalized mentor arrangements, greater use of practical training and assessment, and interdisciplinary platforms. These patterns represent areas of convergence within the selected sample while retaining substantial contextual variation.

#### 3.2.1 Institutionalization of dual-mentorship: building organizational vehicles for boundary-spanning

Boundary-spanning theory draws attention to the organizational separation between academic instruction and frontline practice (37). Across the selected cases, dual-mentor arrangements were a recurring, though not identical, mechanism for involving practitioners in teaching, supervision, and assessment. Their recurrence supports the inclusion of practitioner-engaged mentorship as one component of the analytical framework.

Sichuan University documented an industry-mentor team of more than 120 experts and teaching-development activities for mentors. The Inner Mongolia CDC required practice mentors to hold senior professional titles and at least 5 years of frontline experience. In Shandong, 22 provincial CDC experts were appointed as university master’s supervisors; program records report that the partnership trained 31 postgraduates and co-published 18 papers, including SCI-indexed studies. These outputs illustrate how practitioner participation was formalized through dual-mentorship arrangements across the selected partnerships.

#### 3.2.2 Competency-oriented practical training and assessment

Competency-Based Medical Education (CBME) and Entrustable Professional Activities (EPAs) provide established approaches for linking training to observable professional tasks (38–41). Within the selected cases, several programs supplemented knowledge-based assessment with practical drills or field-performance criteria. The extent and standardization of these changes varied among cases.

Hangzhou’s “teaching-oriented CDC” model included a standardized visual manual linking operational workflows to instruction. Nanjing Medical University and Nanjing CDC established digital experimental teaching and virtual-simulation facilities, while Chongqing incorporated field performance into its integrated training and assessment matrix. Together, these arrangements reflect efforts to make practical expectations more explicit, observable, and responsive to different training contexts.

#### 3.2.3 Integration platforms for clinical medicine, public health, and management: interdisciplinary training networks

Modern public health problems often require contributions from multiple disciplines (42). Consistent with health-ecological perspectives, several education reforms have emphasized connections among clinical medicine, prevention, and management (43). In the selected cases, interdisciplinary platforms were one recurring organizational response, although their scope and operation differed.

Sun Yat-sen University and the Guangzhou CDC established a four-pillar model requiring rotations across public health practice, clinical services, and administrative management. Program documents report 49 students across four cohorts, more than 1,000 h of volunteer epidemiological service, and joint research and policy outputs. These indicators describe implementation reach and interdisciplinary activity across training, service, research, and policy.

By linking training and service activities, these platform-level arrangements seek to reduce departmental boundaries and prepare practitioners to work across clinical intervention, population prevention, and health management. The available documentary evidence describes these organizational arrangements and their associated competency-oriented outputs across the selected cases.

### 3.3 Heterogeneity in model configurations: a multidimensional analysis

Resource Dependence Theory (RDT) provides a lens for examining how organizations obtain and exchange resources they do not control internally (44). From this perspective, APPs can be analyzed as arrangements for managing interorganizational dependencies. The 12 cases were compared across three descriptive dimensions: resource endowments, sources of initiative or authority, and stated training objectives or reported outputs. This framework supports qualitative comparison of heterogeneous development pathways across the selected cases.

#### 3.3.1 Differences in resource endowment: external technology integration versus endogenous asset activation

Given the uneven distribution of resources across regions in China, developed eastern regions and frontier areas adopt distinct resource management strategies.

In metropolitan and coastal settings with greater technological infrastructure, the documents described APPs that incorporated AI, supercomputing, and large-scale surveillance data. Tianjin collaborated with institutions including Beihang University and the National Supercomputing Center on disease-control applications, while Guangzhou established a joint center for disease surveillance and risk assessment. From an RDT perspective, these cases illustrate access to specialized external technological resources and a stated training objective of developing interdisciplinary capacity in digital public health.

In western frontier and ethnic-minority settings, the documents emphasized locally specific practice resources. Dali University worked with public health institutions across eight border prefectures and 31 county-level CDCs, while the Inner Mongolia CDC made zoonotic-disease field settings, a biosafety level-3 laboratory, and response teams available to partner universities. From an RDT perspective, these arrangements illustrate how contextual epidemiological and operational resources can enter interorganizational exchange and support locally responsive workforce development.

#### 3.3.2 Differences in driving forces: administrative authority versus demand-driven initiatives

The cases also differed in how collaboration was initiated and governed. Some networks were established primarily through hierarchical administrative coordination, whereas others were developed in response to operational or teaching needs. These categories describe predominant and potentially overlapping tendencies across the selected cases.

Shandong illustrates a predominantly top-down administrative pathway. Provincial coordination supported the co-construction of a School of Public Health and Health Management by the provincial CDC and Shandong First Medical University, with reciprocal staff appointments and leadership cross-tenure. Institutional isomorphism helps interpret the role of administrative authority in formalizing the structure and coordinating implementation.

Nanjing, Hangzhou, and Shanghai illustrate more demand-responsive pathways shaped by teaching or operational needs. Nanjing developed affiliated training centers through contractual arrangements; Hangzhou linked teaching performance to professional promotion and performance evaluation; and Shanghai used project funding and mentorship in the Qinggeng Program. These cases document comparatively flexible arrangements responsive to teaching and operational needs.

#### 3.3.3 Differences in training objectives: stratified orientation toward academic leadership and applied workforce

Differences in ultimate training objectives reshape curriculum design and evaluation systems across APPs, resulting in a stratified workforce development structure that encompasses both research-oriented leaders and practice-oriented professionals.

Programs involving Sun Yat-sen University and Sichuan University emphasized research-oriented and interdisciplinary skills, including health-data analysis, pathogen tracing, and policy development. The Guangzhou four-pillar model, for example, stated an objective of preparing professionals to contribute to public health decision-making. These statements describe the programs’ orientation and expected post-training contributions.

Programs in Chongqing, Hebei, Inner Mongolia, and Dali placed greater emphasis on preparing an applied workforce for frontline service. Their documented arrangements included integration of MPH and residency training, practice-intensive curricula, regional disease-control settings, and border-area employment pathways. This contrast captures differences in stated emphasis; research-oriented and applied objectives may coexist within individual partnerships.

Drawing on Resource Dependence Theory (RDT), Figure 3 presents a theoretical matrix of differentiated APP trajectories across resource strategy, primary motive, and training endpoint. It contrasts resource-augmenting and context-activating configurations of resource acquisition, organizational coordination, and workforce development.

**Figure 3:**
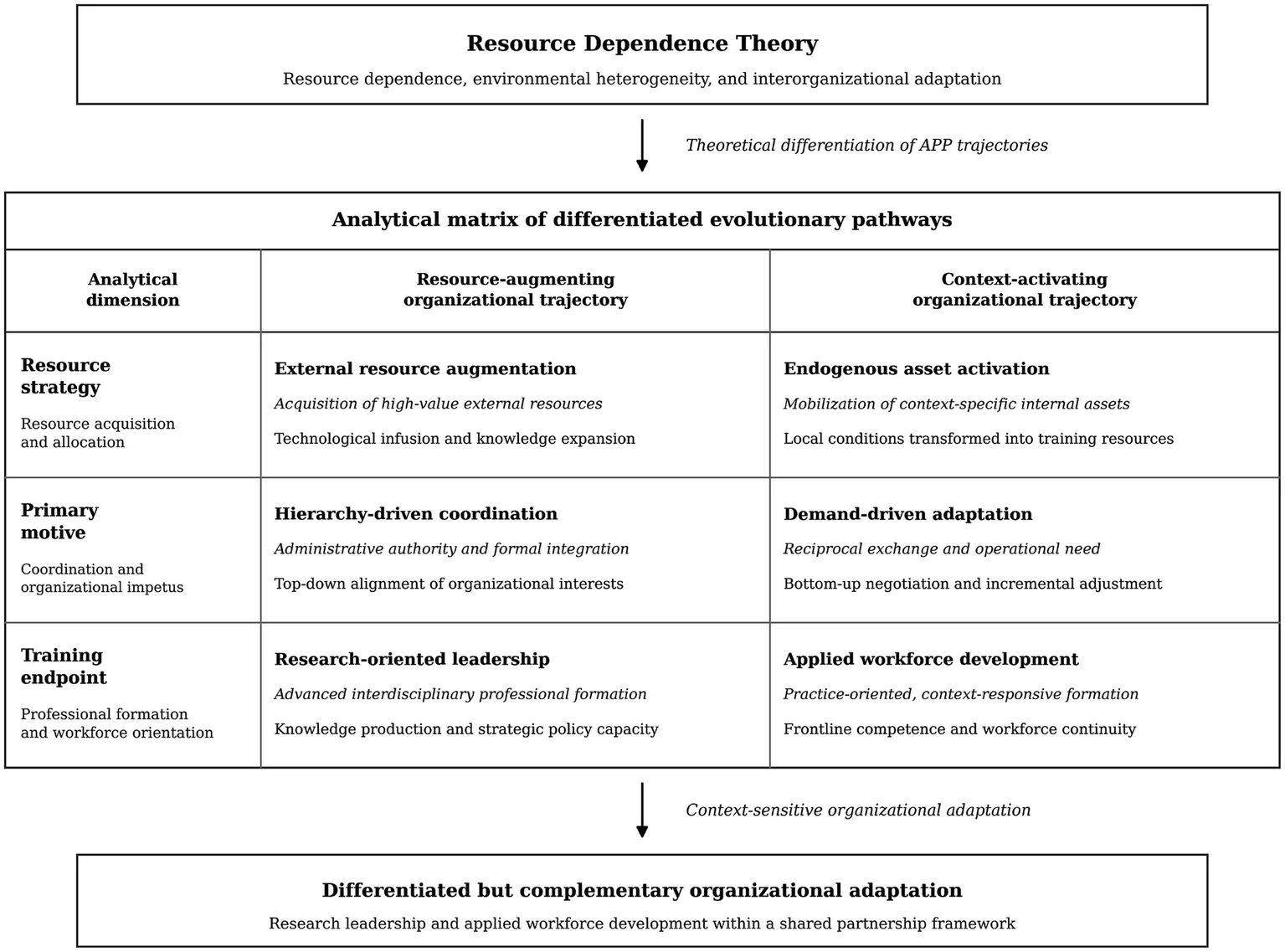
Theoretical matrix of differentiated APP trajectories. Grounded in RDT, the matrix distinguishes two ideal-typical organizational trajectories—resource-augmenting and context-activating—across resource strategy, primary motive, and training endpoint. They represent flexible, potentially overlapping configurations of resource acquisition, organizational coordination, and workforce development.

## 4 Discussion

The comparison of 12 purposively selected cases suggests that APPs in China are shaped by interacting institutional, organizational, and practice-level processes. The four descriptive dimensions can be related to the multilevel theoretical framework, but the mapping is not one-to-one: institutional pressures influence formal arrangements, resource relationships shape organizational choices, and boundary-spanning practices connect training with operational settings. The documentary design supports an interpretive account of how these processes interact across cases.

### 4.1 Macro-level drivers: institutional isomorphism and the restructuring of public health academic relations

This study indicates that the expansion of Academic-Practice Partnerships (APPs) in China is not simply the result of individual educational initiatives, but reflects an institutional response to changing requirements for public health workforce development. Across the 12 cases, collaboration between universities and public health institutions was closely associated with national priorities for strengthening disease prevention and control, integrating education with practice, and improving the capacity of the public health workforce. This pattern is consistent with the concept of coercive isomorphism proposed by DiMaggio and Powell, whereby organizations adopt similar structures in response to regulatory and institutional pressures. In China, such pressures are particularly influential because national policies directly shape the responsibilities, training arrangements, and resource allocation of public health institutions. Recent national initiatives have further mandated collaboration between universities and disease prevention and control institutions, joint development of training resources, and standardized training for public health physicians.

International experience indicates that academic–practice integration can develop through different institutional pathways. In the United States, Academic Health Departments (AHDs) have emerged through formal and informal partnerships negotiated locally. In a 2017 national survey, 376 local health departments provided valid responses, and partnership status was available for 371 departments: 51.6% reported formal AHD partnerships, 21.6% informal partnerships, and 26.7% no partnership. Formal partnerships were associated with higher organizational support for evidence-based decision-making and higher odds of implementing at least one evidence-based chronic-disease intervention (adjusted OR, 2.3; 95% CI, 1.3–4.0) (45). The cross-sectional survey reported these associations, and the accompanying commentary further situates AHDs as locally negotiated bridges between academic and practice settings (46).

Canada and Australia illustrate more institutionally embedded approaches. The Quebec 4P Program established a structured partnership for population-health intervention research training with academic and public health mentorship (47). Program reports described 244 peer-reviewed articles and knowledge-transfer activities, illustrating sustained research and dissemination outputs. Australia’s Master of Applied Epidemiology program, established in 1991, used field-based placements within government health departments to connect training with service and regional capacity development (48). The United Kingdom combines academic and practice-based learning within nationally structured specialty training, while other cross-institutional initiatives have also linked organizations for global health workforce development (49). These examples show that academic–practice integration takes different forms under different governance arrangements.

### 4.2 Meso-level mechanisms: resource dependence and collaborative governance

At the meso level, universities and public health institutions contributed different resources to the documented partnerships (50). Universities commonly provided disciplinary knowledge, educational infrastructure, and analytical capacity, whereas public health institutions provided field settings, surveillance data, and operational expertise. RDT suggests that APPs may help organizations access resources that are difficult to generate independently. The cases illustrate several forms of reciprocal contribution and collaborative governance across different institutional settings.

Resource interdependence also creates organizational risks. The preliminary screening highlighted a continuum from formal agreements to substantive implementation. Where administrative coordination, reciprocal resources, and aligned incentives are weak, partnerships may remain confined to formal agreements. Universities and public health institutions also operate under different performance systems, creating a risk that CDCs become data-collection sites rather than equal workforce-development partners. Sustained APPs therefore appear to require shared decision-making, protected teaching time, transparent resource exchange, and mechanisms for resolving institutional conflict.

### 4.3 Micro-level mechanisms: individual boundary-spanning and competency development

At the micro level, documented mechanisms such as Hangzhou’s battle–teaching integration and Nanjing’s staged practical assessment are intended to connect academic resources with occupational practice. Trainees and mentors may function as boundary spanners by combining methodological knowledge with operational judgment. Comparable field-based approaches are present in Australia’s MAE program and Canada’s Quebec 4P Program. Across these settings, joint supervision and the production of both academic and policy-oriented outputs illustrate how boundary-spanning practices can support two-way knowledge exchange and competency-oriented practice.

These mechanisms require supporting institutional arrangements. Assigning dual-mentorship responsibilities without professional recognition or protected time may increase workload and weaken sustainability. Evidence on workforce evaluation and incentive mechanisms in Chinese CDCs further supports attention to recognition, career development, and organizational support (51). Training models should therefore align field-practice responsibilities with transparent supervision, workload allocation, and performance incentives. At the organizational level, a triple-helix perspective also highlights the importance of coordinated roles across universities, government, and practice institutions (52).

### 4.4 Policy implications and transferability

The integrated framework suggests three areas for policy consideration: formal governance, sustainable resourcing and incentives, and digital public health capacity. These propositions derive from cross-case interpretation and international examples and are intended for context-sensitive implementation and evaluation.

National and provincial health authorities could consider clearer policy recognition of practical teaching and outbreak-response training within disease-control institutions. More formal recognition of frontline practitioners’ educational roles may support continuity beyond short-term administrative agreements. The United Kingdom’s specialty-training arrangements offer one reference point (53), but their relevance to China requires adaptation to local governance and professional systems.

Building on initiatives such as High-Level Schools of Public Health and National Regional Public Health Centers, policymakers could establish tripartite governance committees involving universities, CDCs, and affiliated hospitals. Dual-tutor arrangements offer one mechanism for shared supervision across academic and practice settings (54). Competency-Based Medical Education could be strengthened by incorporating Entrustable Professional Activities—such as outbreak tracing, digital surveillance, and emergency risk communication—into jointly developed evaluation rubrics, drawing on established EPA principles (39–41). These recommendations are best implemented as context-sensitive policy options accompanied by local evaluation.

Participating institutions and local health authorities could also consider stable baseline support for training infrastructure, student field placements, and joint coordination, alongside competitive funding for applied research. Supervisory workload and field mentorship may need explicit recognition in promotion and performance systems. The Quebec 4P initiative provides an international example of structured support (47), but the appropriate funding mix should be assessed locally.

Digital public health is a further area for development. Joint programs could include digital epidemiology, data analysis, and responsible use of artificial intelligence, supported by governance protocols for secure access to practice-based data. The case materials document emerging activity in this area and provide several models that can be adapted to local needs.

The potential transferability of the Chinese experience lies in its mechanisms rather than in direct replication of specific institutional forms. Communities of practice may support shared learning across organizational boundaries (55), while field-based training, joint supervision, and competency-oriented assessment may be useful in different governance settings. Application of China’s administratively coordinated approaches should be adapted to local governance, financing, and workforce conditions. The framework may therefore provide a basis for contextual analysis across varied institutional settings.

## 5 Conclusion

Through comparative analysis of 12 purposively selected cases, this study extends descriptive accounts of university–public health collaboration by offering a four-dimensional typology of institutional, spatial, mechanism-based, and pathway-based arrangements. The findings suggest that APPs in China can be understood as organizational responses to public health governance and workforce-development pressures, while the form and implementation of these partnerships vary across institutional and regional contexts.

### 5.1 Theoretical contributions

The primary conceptual contribution is an integrated multilevel framework linking institutional isomorphism, Resource Dependence Theory, and Boundary-Spanning Theory. The analysis suggests that macro-level institutional pressures shape formal collaboration, meso-level resource interdependence and collaborative governance influence organizational strategies, and micro-level boundary-spanning practices connect training with operational work. The typology may provide a useful basis for examining APPs in other settings, with application guided by local governance structures, financing arrangements, and workforce conditions.

### 5.2 Implications for public policy

For longer-term continuity, policymakers could consider clearer recognition of practical teaching and outbreak-response training, together with stable support for shared training infrastructure and coordination. The balance among legal mandates, administrative guidance, and financial incentives will depend on national and provincial governance arrangements. These options can be introduced through phased feasibility assessment and prospective evaluation.

### 5.3 Recommendations for institutions involved in public health workforce training

Universities and public health institutions could institutionalize collaborative governance through committees involving universities, CDCs, and affiliated hospitals (56). They may strengthen competency-oriented training by incorporating EPAs into jointly designed evaluation processes and by aligning dual-mentor responsibilities with recognition and protected time. Digital components—including epidemiological data analysis, responsible AI use, and digital surveillance—should be introduced with appropriate data-governance, confidentiality, and ethical safeguards. Future research should include mixed-methods evaluations, stakeholder interviews, longitudinal follow-up, and quantitative outcome assessment to examine institutional performance, emergency-response processes, and workforce retention (57).

## 6 Limitations

This study has several limitations. First, purposive selection of 12 established and information-rich cases may favor relatively successful partnerships and under-represent less developed or unsuccessful collaborations; the findings therefore support analytical interpretation rather than statistical generalization. Second, the analysis relied on policy and institutional documents, including authorized internal materials. Such documents may selectively emphasize positive activities and cannot capture the perspectives of trainees, mentors, or managers. Third, although the documents covered 2019–2025, the study did not include standardized longitudinal follow-up or repeated measures of individual partnerships. No independent standardized measures of trainee competency, workforce retention, organizational performance, or population health outcomes were collected. Finally, transferability depends on governance, financing, institutional resources, and workforce conditions. Future research should combine documentary analysis with interviews, observation, longitudinal follow-up, and appropriately designed comparative evaluations.

## Data Availability

The original contributions presented in the study are included in the article/supplementary material, further inquiries can be directed to the corresponding author.
